# Development of an Anatomically Accurate Three-Dimensional-Printed Spine Model From Computed Tomography Data Using Dual-Material Printing for Teaching and Visualization

**DOI:** 10.7759/cureus.98956

**Published:** 2025-12-11

**Authors:** Stefan Tserovski, Todor G Bogdanov, Dimo A Yankov, Vlayko Vodenicharov, Dilyan Ferdinandov

**Affiliations:** 1 Orthopedics and Traumatology, Medical University of Sofia, Sofia, BGR; 2 Medical Physics, Medical University of Sofia, Sofia, BGR; 3 Center of Competence “Clean Technologies for Sustainable Environment – Water, Waste, Energy for Circular Economy”, Sofia University “St. Kliment Ohridski”, Sofia, BGR; 4 Clinic of Neurosurgery, St. Ivan Rilski University Hospital, Sofia, BGR; 5 General Practice, Medical University of Sofia, Sofia, BGR

**Keywords:** anatomical spine model, anatomical visualization, dual-material fabrication, medical education, three-dimensional printing

## Abstract

Accurate anatomical models play a crucial role in medical education by enhancing the understanding of complex spinal structures beyond traditional two-dimensional imaging and cadaveric preparations, which often present limitations in availability and durability. This technical report presents the development of a life-sized, anatomically accurate, three-dimensional-printed spine model derived from computed tomography data. The imaging data were processed and segmented to isolate vertebrae and intervertebral discs, after which refined stereolithography files were generated. The vertebral structures were printed using polylactic acid filament to replicate bone rigidity, while the intervertebral discs were fabricated from thermoplastic polyurethane to simulate their flexibility. The final model preserved precise anatomical proportions, vertebral morphology, and spatial relationships, allowing realistic manipulation and functional visualization of spinal segments. The result is a cost-effective and durable educational tool suitable for teaching, demonstration, and anatomical visualization in medical training. This approach establishes a reproducible workflow for creating high-fidelity, patient-specific models and provides a foundation for future developments, such as incorporating soft tissues or pathological variations.

## Introduction

The human spine is a complex anatomical and mechanical structure that plays a fundamental role in posture, stability, and neuroprotection, making it a central focus in medical education. Traditional teaching methods, such as cadaveric dissection, radiographic imaging, and plastic anatomical models, provide valuable insight but face limitations, including high cost, ethical considerations, tissue degradation, and lack of dynamic mobility or patient specificity [1]. Three-dimensional (3D) printing technologies have introduced new opportunities for creating anatomically accurate and patient-specific spine models derived from computed tomography (CT) imaging, enhancing understanding of spinal anatomy, deformities, and biomechanics [2].

Previous studies have demonstrated that 3D-printed spine models can improve preoperative planning, surgical rehearsal, and intraoperative orientation, particularly in deformity surgery and complex anatomical cases [2,3]. They have been shown to accurately replicate patient anatomy, assist with instrumentation trajectories, and enhance the surgeon’s spatial understanding in cases of congenital scoliosis and severe spine malformations [3]. Other investigations have focused on evaluating the accuracy and surface deviation of printed spine models, demonstrating submillimeter error between printed and digital reconstructions, which confirms their reliability for clinical and educational use [4].

Biomechanical testing has further validated the suitability of 3D-printed vertebrae for surgical simulation. For example, synthetic lumbar vertebrae printed in polylactic acid (PLA) or similar polymers have demonstrated comparable performance to cadaveric bone in pedicle screw insertion torque, axial pullout strength, and stiffness testing [5]. Additionally, studies have shown that combining rigid materials such as PLA for vertebral bodies with flexible thermoplastic polyurethane (TPU) for intervertebral discs provides a more realistic representation of spinal motion and intersegmental flexibility [6].

Our research team previously developed a 3D-printed lumbar spine model from CT data using PLA for vertebrae and TPU for intervertebral discs, primarily for surgical training and simulation of pedicle screw placement [2]. While this demonstrated the feasibility of dual-material fabrication and vertebral biomechanics, the model was limited to the lumbar region and focused on surgical application rather than anatomical education or whole-spine visualization. Other authors and reviews have summarized the growing use of 3D-printed anatomical models and surgical guides in spine care, while dedicated full-scale educational models with functional intervertebral mobility remain less commonly reported [7].

Therefore, there remains a need for a full-scale anatomically accurate spine model that combines rigid vertebral structures with flexible intervertebral discs, designed specifically for educational demonstration and visualization rather than surgical instrumentation. The objective of this study is to describe a reproducible workflow for the development of a complete spine model from anonymized CT data using dual-material 3D printing. The CT dataset was fully anonymized before processing. As no identifiable patient information was used, ethical committee approval was not required according to institutional standards for anonymized imaging data.

## Technical report

CT data of the entire spine (C1 to S1) were obtained using a clinical CT scanner with a slice thickness of 0.625 mm. The images were exported in Digital Imaging and Communications in Medicine (DICOM) format. All personal metadata were removed before processing, resulting in a fully anonymized dataset.

**Figure 1:**
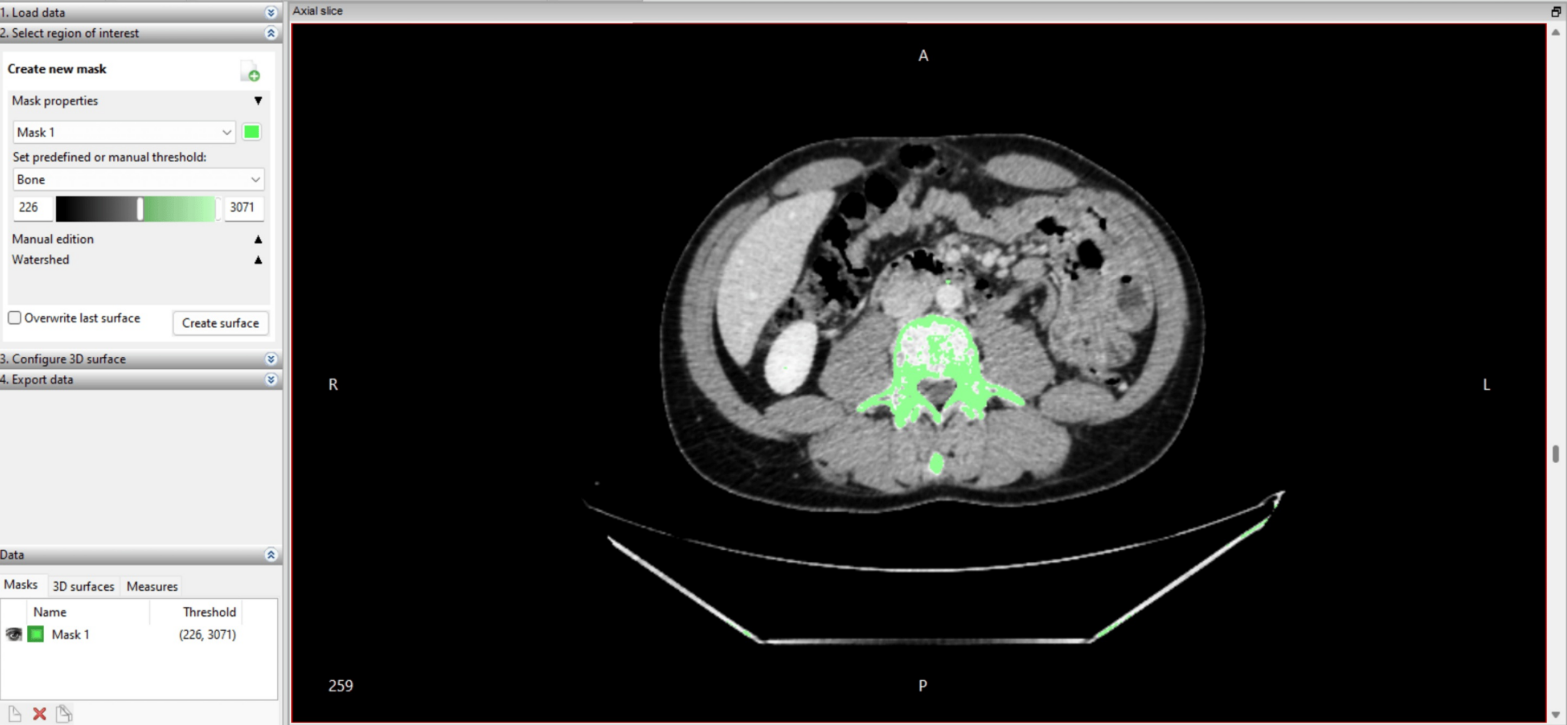
Segmentation interface in InVesalius 3.1, including the thresholding dialog box with the applied Hounsfield range (226-3,071 HU) and the initial three-dimensional reconstruction of the segmented spine.

The DICOM files were imported into InVesalius version 3.1 (Centro de Tecnologia da Informação Renato Archer, Brazil) for segmentation and 3D reconstruction. Bone structures were isolated using the software’s predefined Bone Threshold tool, set between 226 and 3,071 Hounsfield units, which corresponds to cortical and trabecular bone density. This automatic segmentation was followed by manual refinement on axial (Figure 1), sagittal, and coronal views to improve the separation of the vertebral bodies and intervertebral disc spaces, particularly in regions where cortical bone was thin or partially obscured by artefacts.

Artefacts and incomplete segment borders were corrected using slice-by-slice editing tools, region growing, and manual masking. Separate segmentation masks were created for vertebrae and intervertebral disc spaces to facilitate dual-material printing. Once segmentation was complete, 3D surface meshes were generated and exported in stereolithography (.STL) format for further refinement.

The initial stereolithography (.STL) files generated from InVesalius were imported into Meshmixer (Autodesk Inc.) for refinement and preparation for additive manufacturing. Each vertebra was individually inspected, and surface artefacts, irregularities, and segmentation defects were corrected using smoothing, plane cut, and sculpting tools. Sharp edges, incomplete cortical areas, and unwanted noise were removed to ensure structural stability during printing.

Intervertebral discs were not directly segmented from the CT data but were digitally reconstructed to achieve realistic spacing and mobility between vertebrae. Artificial discs were created by inserting cylindrical primitives between adjacent vertebral bodies and sculpting them using the Meshmixer “Sculpt” and “Brush” tools to approximate the natural curvature and anterior-posterior disc profile. The final geometry of each disc was then refined through surface extraction from the inferior endplate of the superior vertebra to match anatomical contouring.

**Figure 2:**
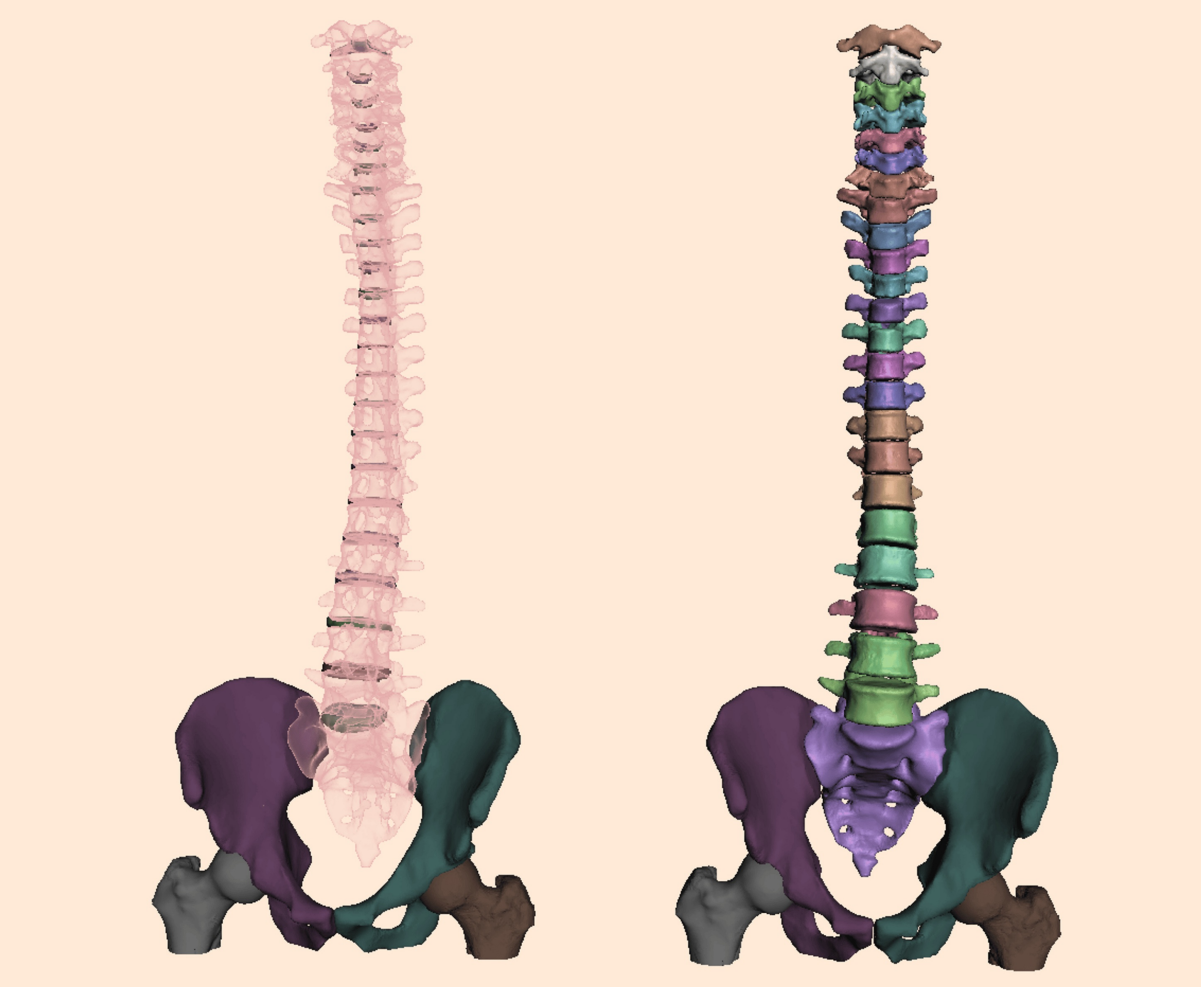
Refined digital spine model. The reconstructed artificial intervertebral discs ( on the left) and the cleaned vertebral bodies (on the right) are displayed, including the addition of the iliac bones and partial femoral segments required for model stabilization.

To provide structural support and enable the creation of a free-standing educational model, the sacrum was digitally fused with bilateral iliac bones and partial proximal femoral structures. A flat plane cut was applied through the iliac crests and femoral shafts to allow the model to stand upright on a stable surface (Figure 2). This modification enables demonstration without external support or mounting systems and allows realistic observation of lumbopelvic alignment.

Vertebral and disc structures were exported as separate STL files to allow dual-material printing in subsequent stages. All models were verified for watertight geometry and non-manifold edges using Meshmixer’s “Inspector” function.

Additive manufacturing was performed using a Bambu Lab X1 Carbon (X1C) fused deposition modeling (FDM) printer. Vertebral elements were fabricated using 3DLine PLA filament in a bone-colored shade to simulate cortical bone appearance. Intervertebral discs were printed separately using 3DLine TPU filament in white, selected for its elastic properties to allow segmental flexibility.

All STL files were sliced using Bambu Studio with high-precision settings. A layer height of 0.12 mm was selected to preserve anatomical detail, particularly in areas such as spinous processes, facet joints, and vertebral endplates. A 0.4 mm brass nozzle was used for both materials. Perimeters were set to four walls, with four top and four bottom solid layers to ensure shell rigidity. Infill density was configured at 15% using a gyroid pattern, providing a balance between strength and material efficiency.

Support structures were generated using tree supports limited to build plate contact only, minimizing post-processing impact on anatomical surfaces. This was particularly advantageous for preserving fine structures on spinous and transverse processes.

To optimize quality and reduce failure risk, vertebrae were individually oriented and distributed across five build plates, with a total printing time of approximately 16 hours. Intervertebral discs were printed in two separate build plates, requiring an additional eight hours of print time. A significant challenge during post-processing involved the removal of flexible TPU supports from the disc surfaces without deforming or tearing the structures.

The pelvic bones and partial proximal femoral segments, required for stability of the free-standing model, were printed from PLA under identical printing parameters on two additional build plates, with a cumulative printing time of 12 hours. This brings the total fabrication time (excluding segmentation and digital modeling) to approximately 36 hours.

Following completion of the printing process, each vertebra and intervertebral disc was manually cleaned of support material. Particular care was taken when processing TPU discs, as their flexibility made them more susceptible to deformation or tearing during support removal. All components were numbered sequentially to preserve anatomical order from C1 to S1. The vertebrae were aligned in anatomical position, and intervertebral discs were inserted between each adjacent vertebral body. Due to the precise digital modelling of disc curvature and endplate conformity, the components were assembled without the need for additional fixation materials such as adhesive, screws, or pins. This maintained both anatomical accuracy and segmental mobility, permitting flexion, extension, and limited rotational movements similar to physiological spinal biomechanics.

To allow the spine to be used as a free-standing educational model, the sacrum was connected to bilaterally printed iliac bones and partial proximal femoral shafts. The iliac and femoral components were printed with an inferior flat plane cut to create a stable base surface. The sacrum was aligned and fitted between the iliac bones, reproducing the natural orientation of the sacroiliac junction. This pelvis-spine unit provided sufficient stability to allow the model to remain upright without external support.

After full assembly, overall spinal curvature was evaluated in the sagittal and coronal planes to ensure physiologically accurate cervical lordosis, thoracic kyphosis, and lumbar lordosis. Each intervertebral disc was tested manually for compressibility and motion response. The model allowed a realistic demonstration of spinal motions, including anterior flexion, extension, lateral bending, and mild axial rotation. It also enabled clear visualization of intervertebral relationships, pedicle orientation, and facet joint alignment, making it suitable for anatomical teaching, biomechanics demonstration, and clinical explanation to students or patients. Although these assessments were qualitative, future studies may incorporate objective parameters, such as quantified range of motion, angular measurements, or dimensional comparison with reference anatomy, to further validate the model’s physiological accuracy. An interactive 3D model of the reconstructed spine, including vertebrae, intervertebral discs, iliac, and partial femoral bones, is available via Sketchfab (Interactive Model 1). The model can be freely explored, rotated, and inspected in high resolution.

**Video 1:** Anatomically accurate three-dimensional-printed spine model – CT-based, polylactic acid/thermoplastic polyurethane.

The final model was used in an academic setting for teaching vertebral anatomy, disc structure, spinal curvatures, and lumbopelvic alignment. During laboratory sessions, students were able to handle the spine directly, disassemble and reassemble individual vertebrae, and examine key landmarks such as pedicles, transverse processes, facet joints, and intervertebral endplates. Instructors used the model to demonstrate the mechanics of spinal curvature, the effect of disc height on foraminal dimensions, and the changes in vertebral alignment that occur in conditions such as scoliosis or segmental instability. The ability to tilt or rotate specific vertebrae helped illustrate concepts such as coupled motion in the cervical spine and thoracolumbar transition mechanics. Unlike rigid plastic or cadaveric models, this spine can be manipulated dynamically, is lightweight, durable, and easily reproducible, making it suitable for repeated hands-on teaching sessions and clinical explanations to learners.

## Discussion

The assembled model was used in undergraduate and postgraduate medical education to demonstrate vertebral morphology, spinal curvature, intervertebral disc structure, and lumbopelvic alignment. Students were able to physically manipulate the spine, observe flexion and extension, and disassemble individual vertebrae to study anatomical landmarks such as pedicles, laminae, facet joints, neural foramina, and endplates. Unlike traditional rigid anatomical models, this dual-material design provided both structural accuracy and functional mobility. The detachable nature of the vertebrae and discs enabled instructors to demonstrate changes in vertebral alignment and intersegmental relationships that are characteristic of clinical conditions such as scoliosis or spondylolisthesis. Although the absence of soft tissues prevents reproduction of neural or ligamentous components of these disorders, the model still allows clear visualization of the bony anatomy and mechanical principles underlying these pathologies. Similar improvements in educational engagement and spatial understanding have been reported in previous studies utilizing 3D-printed spinal or skeletal models for medical training [3,5,7].

The model was also applied in clinical teaching sessions to support the explanation of spinal disorders to patients and trainees. Its life-size scale, anatomical fidelity, and realistic motion allowed clear visualization of biomechanical relationships and pathological changes. These findings are consistent with earlier work demonstrating that 3D-printed spine replicas enhance both surgical planning and patient communication through improved tactile and visual comprehension [2,4,6]. Surgeons reported that the model was particularly useful for communicating the mechanics of spinal instability and the role of intervertebral discs during movement.

The fabrication approach used in this study offers several interconnected advantages. Because the model is derived directly from CT data, it maintains patient-specific anatomy while remaining reproducible. The use of dual-material printing with PLA for vertebrae and TPU for discs provides both rigidity and elasticity, achieving realistic intervertebral motion. The modular architecture enables partial disassembly for detailed examination of vertebral landmarks, while the use of cost-effective filaments allows affordable large-scale production. Compared with cadaveric specimens, the printed model is durable, lightweight, and reusable, requiring no special preservation or ethical approval. Furthermore, the digital file can be freely shared, modified, and reprinted, and an interactive version is available on Sketchfab as Interactive Model 1, allowing global access to the model for educational use.

Despite its anatomical accuracy, several limitations exist. The model does not include soft tissues such as ligaments, muscles, dura mater, or nerve roots, limiting its use in neurosurgical simulation. The elastic properties of TPU discs approximate only gross motion and do not fully replicate the viscoelastic behavior of real intervertebral discs. The sacroiliac joint is fixed to allow model stability, and no motion simulation is present in this region. Furthermore, removal of support material from flexible TPU components was technically challenging and risks disc deformation if not performed carefully.

Future developments may incorporate ligamentous structures, colored nerve roots, or a 3D-printed spinal cord to improve neurosurgical relevance. Integration of magnets or snap-fit mechanisms could allow easier assembly and disc replacement. Adding pathological datasets (e.g., scoliosis, disc degeneration, trauma) would enhance clinical applicability, and inclusion of soft tissues using silicone or elastomeric polymers may further improve biomechanical realism. Quantitative validation, including dimensional accuracy and surface deviation measurements, may also be included in future work, as these assessments were beyond the scope of the present technical report.

## Conclusions

This technical report presents a complete workflow for developing an anatomically accurate, life-size, 3D-printed model of the human spine using anonymized CT data and dual-material additive manufacturing. By combining rigid PLA vertebrae with flexible TPU intervertebral discs, the model successfully reproduces both the structural morphology and functional mobility of the spinal column. The integration of the pelvis and proximal femurs enables free-standing demonstration, enhancing its value as an educational tool. The resulting model is cost-effective, reusable, anatomically realistic, and suitable for hands-on teaching, anatomical visualization, and patient communication. Unlike conventional rigid anatomical models or cadaveric specimens, it offers modular disassembly and real intervertebral motion without ethical constraints. Although soft tissues and detailed biomechanics are not fully replicated, the method is reproducible and can be expanded to include ligaments, nerves, or pathological anatomy in future iterations. Objective biomechanical and dimensional validation will be required in subsequent studies to fully assess the physiological accuracy of the model. This approach provides an accessible bridge between medical imaging and physical anatomical education and demonstrates the potential of 3D printing to enhance spine teaching and clinical understanding.
